# The effect of growth hormone on ovarian function recovery in a mouse model of ovarian insufficiency

**DOI:** 10.3389/fendo.2023.1184977

**Published:** 2023-10-03

**Authors:** Su Mi Kim, Jung Young Yoo, Yeon Hee Hong, Jaewang Lee, Ji Hyang Kim, Jung Ryeol Lee

**Affiliations:** ^1^ Department of Obstetrics and Gynecology, Chungbuk National University Hospital, Cheongju, Republic of Korea; ^2^ Department of Obstetrics and Gynecology, College of Medicine, Seoul National University, Seoul, Republic of Korea; ^3^ Department of Obstetrics and Gynecology, Seoul National University Bundang Hospital, Seongnam, Republic of Korea; ^4^ Department of Biomedical Laboratory Science, Eulji University, Seongnam, Republic of Korea; ^5^ Department of Obstetrics and Gynecology, Fertility Center of CHA Bundang Medical Center, College of Medicine, CHA University, Seongnam, Republic of Korea

**Keywords:** growth hormone, ovarian insufficiency, cyclophosphamide, breast cancer, ovarian regeneration, fertility preservation

## Abstract

**Objectives:**

To evaluate the effects and mechanisms of action of growth hormone (GH) in the recovery of ovarian function in ovarian insufficiency induced by cyclophosphamide (CP) in a mouse model.

**Materials and methods:**

After inducing ovarian insufficiency by administering 400 mg/kg of CP intraperitoneally to 6-week-old ICR mice, the mice were divided into four groups (control, CP, 1 mg/kg GH, and 2 mg/kg GH) with 10 mice in each group. GH was administered a week later for 7 days. Five mice from each group were sacrificed the next day, and their ovaries were collected for histological examination. The remaining mice were superovulated for *in vitro* fertilization (IVF). The terminal deoxynucleotidyl transferase dUTP-nick end labeling assay was performed to detect apoptosis. Masson’s trichrome staining was used to analyze the degree of fibrosis. To quantify angiogenesis, CD31 immunohistochemistry was performed. Angiogenesis-related gene expression profiles were assessed using quantitative reverse transcription polymerase chain reaction.

**Results:**

CP induced the loss of non-growing (primordial and primary) follicles while GH significantly protected primordial follicles and increased follicular quality. The CP group showed a decrease in fertilization and blastocyst formation rates in IVF. In contrast, the GH treatment group showed dose-dependent enhanced IVF outcomes. Furthermore, GH treatment decreased apoptosis and stromal fibrosis and increased angiogenesis. Many genes involved in angiogenesis, especially Leptin (*Lep*), platelet endothelial cell adhesion molecule 1 (*Pecam-1*), and angiogenin (*Ang*) were up-regulated in the GH treatment groups.

**Conclusion:**

GH treatment may promote the recovery of ovarian function in ovarian insufficiency induced by the administration of CP via decreasing apoptosis and stromal fibrosis and upregulating *Lep*, *Pecam-1*, and *Ang* genes.

## 1 Introduction

Cancer is one of the major causes impacting health globally, and the affected populations includes young adults (1). The incidence of cancer in 20-39-year-old women has been reported to be 57.0 per 100,000 (2). With the improving survival rate among patients with cancer, the need to improve the quality of life among cancer survivors is also increasing. Fertility preservation is an important issue for cancer survivors (1). Moreover, the demand for fertility preservation among cancer survivors is expected to increase, given the increasing postponement of marriage and childbearing among women worldwide.

Cyclophosphamide (CP) is an alkylating agent widely used to treat various malignancies, including breast cancer and multiple myeloma. It is associated with a high risk of gonadotoxicity, as it can cause apoptosis, overactivation of primordial follicles, and atresia of growing follicles (3). Administration of CP could induce premature ovarian insufficiency and cause cognitive dysfunction, osteopenia, and cardiovascular disease (4).

Oocyte and embryo cryopreservation are the standard methods of fertility preservation; however, they cannot be used to recover ovarian endocrine function (5). Ovarian tissue cryopreservation can be used to restore ovarian endocrine function (6); however, it introduces the possibility of tumor cell re-entrance after transplantation of the cryopreserved tissue in patients with cancer (7). Moreover, these cryopreservation methods are not possible in places where assisted reproductive centers do not exist. In such cases, the option of using a gonadotropin-releasing hormone agonist (GnRHa) could be offered to young women with breast cancer as an alternative. The evidence for using GnRHa to preserve function is conflicting; as such, this method cannot currently replace established methods (8). Meanwhile, research on therapeutic agents with the potential to restore damaged ovarian function has been conducted recently. One such therapeutic agent involves stem cells. However, the safety of using stem cells as therapeutic agents has not yet been proven, and the methods for their injection have not yet been standardized (9).

Growth hormone (GH) is a monomeric peptide hormone mainly secreted by the anterior pituitary gland (10). Classically, pituitary GH was understood to act on its hepatic receptors to produce hepatic insulin-like growth hormone I (IGF-I). However, this view has been revised; both the liver and several peripheral organs, including the ovaries, are now understood to produce IGF-I through GH stimulation. Moreover, reproductive cells can produce GH to control their own signaling pathways via paracrine or autocrine actions (11). GH plays essential roles in cell growth and development and metabolism throughout the body. In female reproduction, GH is required for the onset of puberty, follicular development, steroidogenesis, and oogenesis in the ovaries (12). Due to its involvement in the regulation of female infertility, GH has received the most attention among adjuvant therapies for *in vitro* fertilization (IVF), especially in patients with poor ovarian response (POR) (13). As per a recent systematic review and meta-analysis, GH supplementation in patients with POR increased the clinical pregnancy rate, number of retrieved oocytes, number of metaphase II (MII) oocytes, and live birth rate (14, 15).

GH has been administered during IVF clinically, and it appears safer than are other therapeutic agents, such as stem cell therapy; however, its ability to restore ovarian function has not yet been clarified. Only a few studies have explored the effects of GH in the recovery of ovarian function using animal models. Mahran et al. reported that GH has a radioprotective effect, and it rescued ovarian function in a rat model (16). Liu et al. showed that GH promotes ovarian tissue repair, estrogen release, and oocyte maturation via activation of the Notch-1 signaling pathway in a mouse model (17). Yigiter et al. found that GH reduced ovarian tissue damage in a rat model; this may be attributed to the antioxidant properties of GH (18). Nevertheless, little is known about the mechanisms by which GH restores ovarian function. Therefore, the effects of GH and the mechanisms by which it promotes ovarian function recovery need to be evaluated.

The present study was designed with the aim of evaluating the effects and mechanisms of the action of GH on ovarian function recovery in ovarian insufficiency (OI) induced by the administration of CP in a mouse model.

## 2 Materials and methods

### 2.1 Animals

Six-week-old female ICR mice (Orient Bio Inc., South Korea) were chosen to establish an animal model. ICR mice have widely been used in animal model experiments using CP (19, 20). They were acclimated for one week before experimentation and were housed in ventilated cages at room temperature in a humidity- and light-controlled environment with 12-hour light-dark cycles. They were provided with food and water ad libitum.

### 2.2 Study design

A preliminary study was conducted to determine the dose of CP (Sigma-Aldrich, Burlington, MA, USA) to be administered to induce OI (**Supplementary Figures 1-3**). The mice were divided into three groups (four mice per group) as follows: 1) the control group, mice receiving only 200 μL of 0.9% normal saline injection; 2) the low-dose CP group, mice receiving a single intraperitoneal injection of 400 mg/kg CP; and 3) the high-dose CP group, mice receiving an intraperitoneal injection of 400 mg/kg CP every 2 days for a total of 6 days (a total of 1200 mg/kg of CP was injected). Three days after the last injection, ovaries were collected to perform hematoxylin and eosin (H&E) staining, terminal deoxynucleotidyl transferase dUTP nick-end labeling (TUNEL) assay, and Masson’s trichrome staining. The high-dose CP group showed a high mortality rate (50%). Furthermore, the primordial follicle damage (**Supplementary Figure 1**), apoptosis rate (**Supplementary Figure 2**), and fibrosis rate (**Supplementary Figure 3**) were too high to be analyzed. We concluded that the low-dose CP group achieved the desired degree of ovarian damage, as this produced no apparent harm to the mice except for the deterioration of ovarian function.

**Figure 1:**
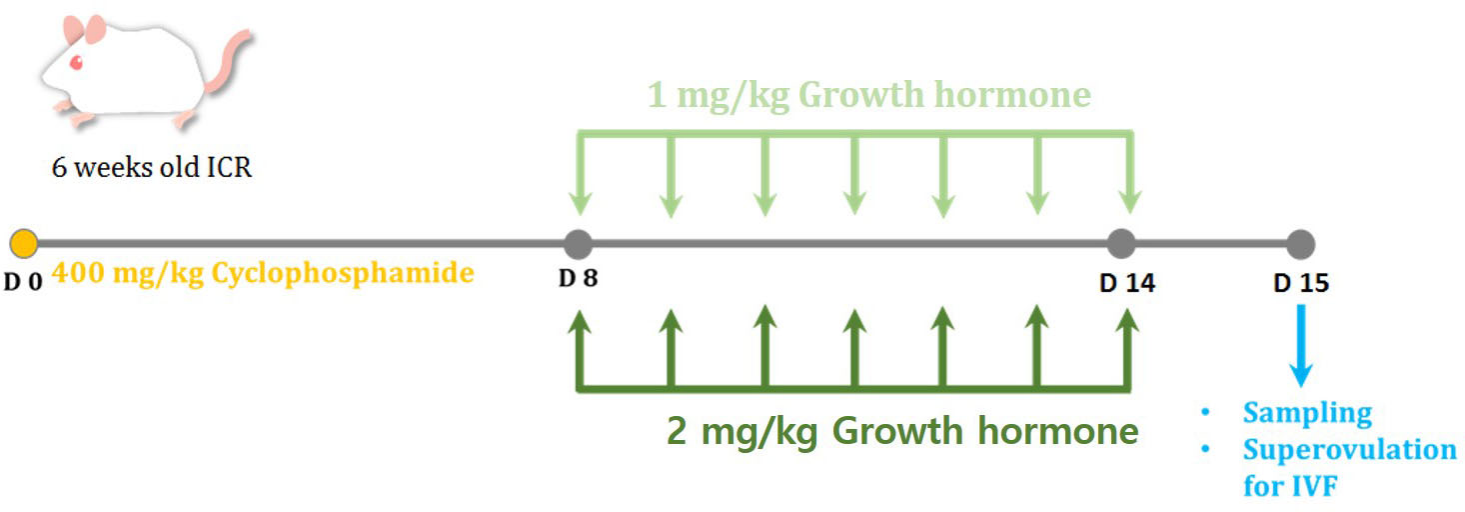
The experimental scheme for the present study. To induce ovarian insufficiency (OI), 400 mg/kg of cyclophosphamide (CP) was administered in a single intraperitoneal injection. The mice were randomly divided into four groups: control, CP, growth hormone (GH) 1-mg/kg-treated, and GH 2-mg/kg-treated groups. Normal saline or GH was administered a week after induction of OI for 7 days. All mice were sacrificed a day after the last injection of GH.

**Figure 1** shows an overview of the methodology of the present study. A new set of mice were randomly divided into four groups (10 mice per group) as follows: 1) the control group, mice receiving 200 μL of 0.9% normal saline injection; 2) the CP group, OI mice administered a single intraperitoneal injection of 400 mg/kg CP; 3) low-dose GH treatment group, OI mice injected with 1 mg/kg GH (Growtropin, DONG-A ST, Seoul, Korea); and 4) high-dose GH treatment group, OI mice injected with 2 mg/kg GH. Normal saline or GH was administered intraperitoneally a week after induction of OI for 7 days. The dose (16, 17) and route of GH were determined based on the recommendation of previously published studies administration (18, 21). Five mice from each group were utilized for histological examination, while the other five from each group were superovulated for IVF. Subsequently, all mice were sacrificed a day after the last injection of GH.

### 2.3 Histological analysis

Ovaries were fixed in formalin and embedded with paraffin. To count the number of follicles and evaluate follicular development and quality, serial tissue sections of 5-µm thickness were stained with H&E solution (Cancer Diagnostics, Durham, NC, USA) and visualized using light microscopy (Nikon, Tokyo, Japan). The total numbers of follicles less than 48 µm in diameter were counted in at least eight selected non-overlapping fields. Follicles were classified into the following developmental stage categories (22): (1) primordial follicles, a single layer of flattened granulosa cells; (2) primary follicles, a single layer of granulosa cells of which one or more were cuboidal; (3) secondary follicles, two or more layers of cuboidal granulosa cells lacking an antral space; (4) antral follicles, multiple layers of cuboidal granulosa cells with an antrum. The follicular quality was assessed using the following morphological criteria (23, 24): (1) primordial and primary follicles: grade 1, spherical with even distribution of granulosa cells; grade 2, granulosa cells pulled away from the edge of the follicle but with spherical oocyte; grade 3, granulosa cells with pyknotic nuclei and misshapen oocyte; (2) secondary and antral follicles: grade 1, evenly distributed granulosa and theca cells with small spaces between cells and a spherical oocyte; grade 2, intact theca cells, apparent loss of granulosa cells but with the oocyte remaining spherical; grade 3, disrupted granulosa cells with theca cells pulled away from the edge, with pyknotic nuclei and a misshapen vacuolated oocyte.

### 2.4 *In vitro* fertilization

Five mice from each group were hyperstimulated via intraperitoneal injection of 7.5 IU of serum gonadotropin from a pregnant mare, followed by an injection of 7.5 IU of human chorionic gonadotropin after 48 hours. After 16 hours, the mice were sacrificed, and the cumulus-oocyte complexes were harvested via fallopian tube puncture. The epididymal sperms were retrieved from 10-week-old male ICR mice, and the sperm suspensions were incubated at 37°C in humidified 5% CO_2_ air for an hour. The metaphase II oocytes were inseminated with sperms and incubated at 37°C in humidified 5% CO_2_ air for 4 hours. Inseminated oocytes were washed with HTF medium (FUJIFILM Irvine Scientific, Santa Ana, CA, USA) and incubated in a fresh HTF medium. Fertilization was assessed based on the division of the zygote into a two-cell embryo 24 hours after insemination. Subsequently, the cleaved embryos were transferred to KSOM media (MilliporeSigma, Burlington, MA, USA). Blastocyst development was assessed 72 hours after insemination.

### 2.5 Analysis of ovarian apoptosis

Apoptosis of the ovarian tissue was analyzed using the TUNEL assay (Promega, Madison, WI, USA). In summary, after deparaffinization and rehydration, the slides were briefly treated with proteinase K solution (Promega) for 20 minutes at room temperature, followed by incubation with a TUNEL reaction mixture for an hour at 37°C. After washing with D-PBS, the slides were mounted with Vectashield mounting medium with 4’,6-diamidino-2-phenylindole (DAPI, ImmunoBioSience Corp., Mukilteo, WA, USA) and examined under an inverted Zeiss AX10 microscope. Apoptotic cells were visualized as green fluorescence, and DAPI-stained cells were visualized as blue fluorescence. Follicles containing more than 30% apoptotic cells were regarded as apoptotic follicles (25). The apoptotic areas were quantified using the Image J software (National Institutes of Health, Bethesda, MD, USA). The apoptosis ratio (%) was defined as the ratio of TUNEL-stained area per the total ovary area.

### 2.6 Analysis of ovarian stromal fibrosis

Masson’s trichrome staining was performed to analyze ovarian stromal fibrosis using the Roche Trichrome III Blue Staining Kit (Roche, Basel, Switzerland). After deparaffinization and rehydration, the slides were treated with Bouin solution (Sigma-Aldrich) for an hour at 60°C. After washing with distilled water, the slides were stained with Weigert’s iron hematoxylin solution for 10 minutes. The slides were then washed with distilled water and were placed in Biebrich-scarlet acid fuchsin solution for 5 minutes, followed by washing with distilled water. The slides were then placed in an aniline blue solution for 8 minutes. After washing with distilled water, the slides were treated with 0.5% acetic acid for one minute. The slides were then dehydrated in ethanol and treated with xylene for 1 minute. Finally, the slides were mounted with a mounting medium (Dako). Stained slides were analyzed using the Image J software. The fibrotic area (%) was defined as the ratio of Masson’s trichrome-stained area per the total ovary area.

### 2.7 Immunohistochemistry for CD31

The density of the blood vessel was evaluated through immunohistochemistry with anti-CD31 antibodies. After deparaffinization and rehydration, target antigen retrieval was performed using citrate buffer. The slides were placed in a microwave oven and heated for 20 minutes at 700 W, followed by cooling for 30 minutes at room temperature. The slides were placed in a peroxidase-blocking solution for 10 minutes and incubated overnight at 4°C with an anti-CD31 antibody (1:200 dilution, Abcam, Cambridge, UK). After washing, the slides were treated with EnVision+HRP solution (Dako) for 60 minutes and then the substrate-chromogen solution (1:50, Dako) for 10 minutes. The slides were counterstained with hematoxylin and dehydrated with ethanol and xylene. The CD31-positive areas were determined using the Image J software (National Institutes of Health). The CD31-positive area (%) was defined as the ratio of the CD31-stained area per the total ovary area.

### 2.8 Western blot for CD31

Protein samples obtained from mouse ovaries were incubated with PRO-PREPTM Protein Extraction Solution (iNtRON Biotechnology, Seongnam, South Korea). Twenty micrograms of proteins were separated using 10% sodium dodecyl-sulfate polyacrylamide gel electrophoresis and transferred to a polyvinylidene difluoride membrane using a Mini Trans-Blot cell and criterion blotter (Bio-rad, Hercules, CA, USA). After blocking with 5% skim milk in 1× TBST with 0.1% Tween 20 (Sigma-Aldrich) at room temperature for 1 hour, the blots were probed at 4°C overnight with a 1:300 dilution of anti-CD31 primary antibodies (BS-0195R; Bioss, Woburn, MA, USA) and a 1:1000 dilution of anti-β-tubulin (s2128; Cell Signaling Technology, Danvers, MA, USA). This was followed by incubation in a 1:2000 dilution of mouse anti-rabbit IgG secondary antibody (sc-2357; Santa Cruz Biotechnology, Dallas, TX, USA) at room temperature for 1 hour. Band intensity was quantified using Image J and normalized to β-tubulin band intensity.

### 2.9 Quantitative reverse transcription-polymerase chain reaction and data analysis

Angiogenesis-related gene expression profiles were assessed using the quantitative reverse transcription polymerase chain reaction (RT-qPCR). Total RNA was extracted from the ovarian tissues using TRizol Reagent (Invitrogen, Carlsbad, CA, USA) according to the manufacturer’s protocol. cDNA synthesis and the subsequent real-time PCR were carried out using the AccuTarget™ qPCR Screening Kit (Bioneer, Daejeon, South Korea). The real-time PCR reaction mixture contained a final volume of 20 μL, with 1 μg reverse-transcribed total RNA and a 2×PCR master mix. All qRT-PCR amplifications and analyses were conducted using an Accupower qPCR PreMix kit in an Exicycler 96 Real-Time Quantitative Thermal Block (Bioneer). The PCR conditions were as follows: initial denaturation of the template DNA at 95°C for 10 minutes, followed by 40 cycles of amplification of the template DNA, each cycle of which consisted of denaturation at 95°C for 5 seconds and primer annealing and extension at 58°C for 25 seconds. All reactions were performed in triplicate. The details of the analyzed genes are listed in **Supplementary Table 1**. Data analysis was performed using the relative quantitative method, and the ΔΔCT value was used to determine the relative fold change in gene expression. Scatterplots of the mRNA sequences that were upregulated or downregulated by 1-fold compared to those of the control group were created. Heatmap results were generated using the R software (R Foundation for Statistical Computing, Vienna, Austria). All the data were normalized to the reference β-actin (*ACTB*) gene expression level.

### 2.10 Statistical analysis

The data were presented as mean ± standard error of the mean. Multiple comparisons were performed using one-way analysis of variance, followed by Tukey’s method as a *post-hoc* test, using GraphPad Prism, version 8.4.0 (Graphpad Software Inc., San Diego, CA, USA). Statistical significance was set at P <0.05.

## 3 Results

### 3.1 Gross observation and ovarian weight changes

**Figure 2:**
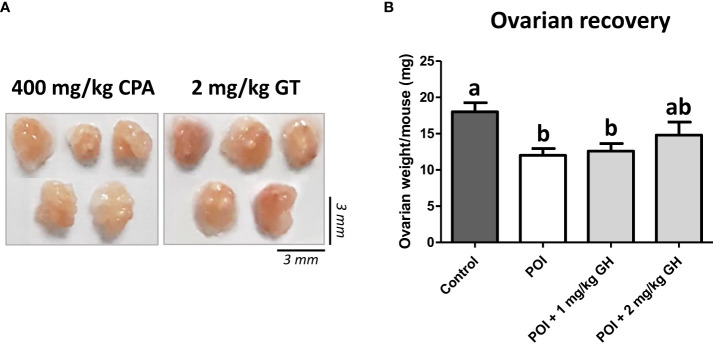
**(A)** The gross appearance of the ovaries. **(B)** The average ovarian weight. Data are presented as mean ± SEM; different superscript letters indicate statistically significant differences (*p* < 0.05). CP, cyclophosphamide; GH, growth hormone.

No significant side effects of GH were observed during the experiments. One day after GH administration, ovaries were harvested, and their weights were determined. Upon gross observation, the ovaries appeared to be greater in size in the GH-treated group than in the CP group (**Figure 2A**). As indicated in **Figure 2B**, ovarian weight was significantly decreased after CP administration. The ovarian weight increased in the GH-treated group but not to a statistically significant level.

### 3.2 Evaluation of ovarian follicle morphology

**Figure 3:**
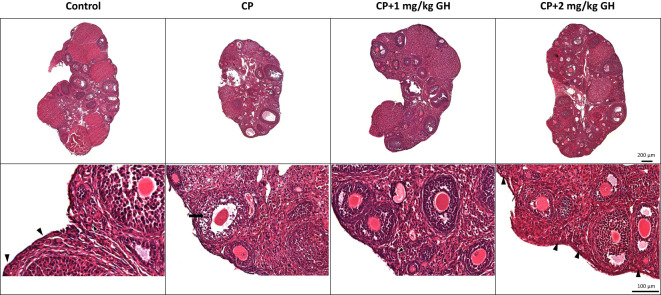
Representative images of hematoxylin and eosin-stained ovarian tissue. The arrow head indicates promordial follicles, and the arrow indicates a grade 3, atretic follicle. CP, cyclophosphamide; GH, growth hormone.

**Figure 4:**
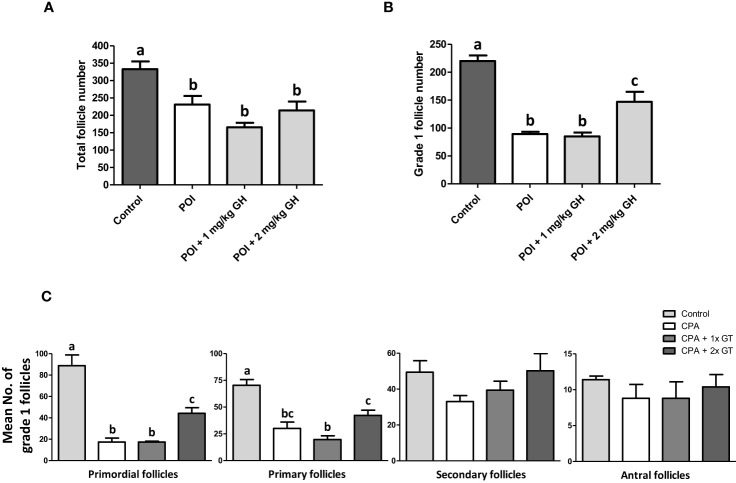
Histological analysis of ovaries. **(A)** Total follicle number, **(B)** grade 1 follicle number, and **(C)** mean numbers of grade 1 follicles according to the follicular development stages of ovarian tissue. Data are presented as mean ± SEM; n=5; different superscript letters indicate statistically significant differences (*p* < 0.05). CP, cyclophosphamide; GH, growth hormone.

The number and structural quality of follicles were evaluated using H&E-stained ovaries (**Figure 3**). The stromal density and normality of ovarian follicles were decreased in the CP group. However, the density of stromal cells in GH-treated groups was higher than that in the CP group, and relatively high-graded follicles were observed in the GH-treated groups. The total number of follicles and the number of grade 1 (G1) follicles decreased in the CP and GH-treated groups compared to those in the control group (**Figures 4A, B**). A statistically significant difference in the total number of follicles was not observed between the CP and GH-treated groups (**Figure 4A**); however, the number of G1 follicles was significantly increased in the 2 mg/kg GH-treated group compared with that in the CP group (p <0.05) (**Figure 4B**). **Figure 4C** represents the mean G1 follicle number according to follicular development stages. The numbers of primordial and primary follicles were dramatically decreased in the CP group (p <0.05), indicating that CP induces non-growing follicle loss. The number of G1 primordial follicles was significantly increased in the 2 mg/kg GH-treated group compared to that in the CP group. The number of G1 primary and secondary follicles in the GH-treated groups was increased compared with those in the CP group, but this increase was not statistically significant. Thus, GH treatment prevented the primordial follicle loss induced by CP administration and improved follicular quality.

### 3.3 *In vitro* fertilization

**Figure 5:**
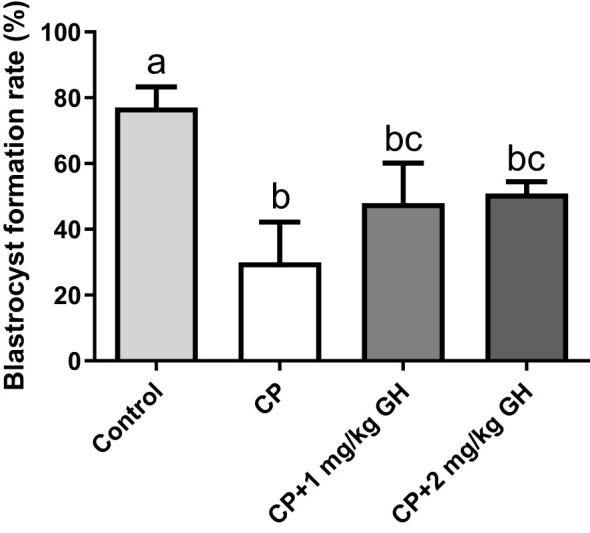
Bar graphs depicring the blastocystformation rate. Data are presented as mean ± SEM; n=5; different superscript letters indicate statistically significant differences (*p* < 0.05). CP, cyclophosphamide; GH, growth hormone.

**Table 1** shows the IVF data. **Supplementary Figure 4** depicts the proportion of retrieved oocytes that developed into blastocysts on day 4. The MII oocyte level was 88.9% in the control group but only 78.8% in the CP group. Meanwhile, the MII oocyte levels were 81.1% and 89.4% in the 1 mg/kg GH- and 2 mg/kg GH-treated groups, respectively. After insemination, the fertilization and blastocyst formation rates were also decreased in the CP group (81.7% and 26.9%, respectively), whereas the corresponding values were 88.3% and 81.3%, respectively, in the control group. The fertilization and blastocyst formation rates were increased in the GH-treated groups: 91.0% and 40.5%, respectively, in the 1 mg/kg GH-treated group and 92.3% and 50.0%, respectively, in the 2 mg/kg GH-treated group. The blastocyst formation rate was significantly decreased in the CP group. In contrast, the blastocyst formation rate showed a dose-dependent increase in the GH-treated groups (**Figure 5**), with the rates not differing significantly from that in the control group, thereby indicating a restoration of fertility to a level similar to that of the control group.

**Table 1:** IVF outcomes with the control group, CP group, and GH-treated groups.

Group	No. of mouse	No. of normal oocyte	Mean No. of normal oocyte	No. of MII oocyte	Maturation rate (%)	Fertilization rate (%)	Blastocyst formation rate (%)
Control	5	135	27	120	88.9	88.3	81.3
CP	5	104	20.8	82	78.8	81.7	26.9
CP+GH 1mg/kg	5	164	32.8	133	81.1	91.0	40.5
CP+ GH 2mg/kg	5	160	32	143	89.4	92.3	50.0

CP, cyclophosphamide; GH, growth hormone.

### 3.4 Evaluation of apoptosis and fibrosis

**Figure 6:**
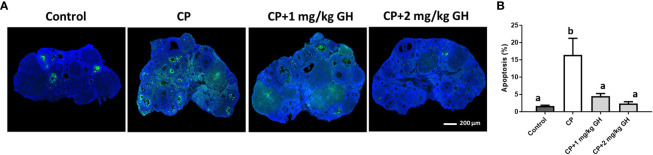
Terminal deoxynucleotidyl transferase dUTP nick-end labeling (TUNEL) assay. **(A)** Green fluorescence indicates apoptotic cells and blue fluorescence (DAPI) indicates intact cell nuclei. **(B)** Quantification of the TUNEL-positive area. Data are presented as mean ± SEM; n=5 and different superscript letters indicate statistically significant differences (*p* < 0.05). DAPI: 4’,6-diamidino-2-phenylindole CP, cyclophosphamide; GH, growth hormone.

**Figure 7:**
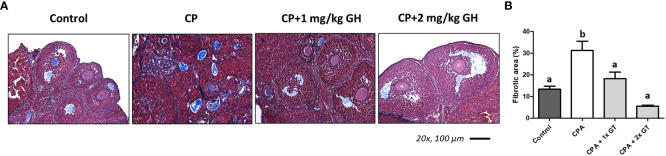
Masson’s trichrome stain. **(A)** The fibrotic surface, nuclei, and cytoplasm, stained blue, black, and red, respectively. **(B)** Quantification of the fibrotic area. Graphs are presented as mean ± SEM; n=5 and different superscript letters indicate statistically significant differences (*p* < 0.05). CP, cyclophosphamide; GH, growth hormone.

**Figure 6A** shows a representative image of TUNEL-positive areas in ovaries from each group. Apoptosis was significantly increased in the CP group compared to that in the GH-treated groups and showed dose dependence (**Figure 6B**). **Figure 7A** shows a representative image of Masson’s trichrome-stained ovarian tissue. This staining is used to detect collagen fibers indicating tissue fibrosis (26). The CP group sample showed an extensive fibrotic area, while the fibrotic areas and their proportions were significantly reduced in the GH-treated groups (**Figure 7B**).

### 3.5 Evaluation of angiogenesis and related gene expression

**Figure 8:**
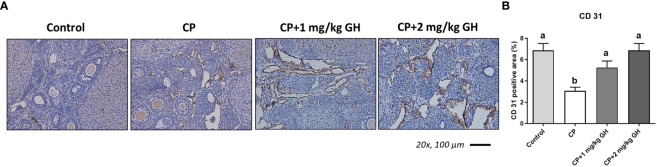
Immunohistochemical staining of ovarian tissue with CD31. **(A)** Brown-stained cells are CD31-positive. **(B)** Quantification of the CD31-positive area in ovarian tissues. Data are presented as mean ± SEM; n=5; different superscript letters indicate statistically significant differences (*p* < 0.05). CP, cyclophosphamide; GH, growth hormone.

**Figure 9:**
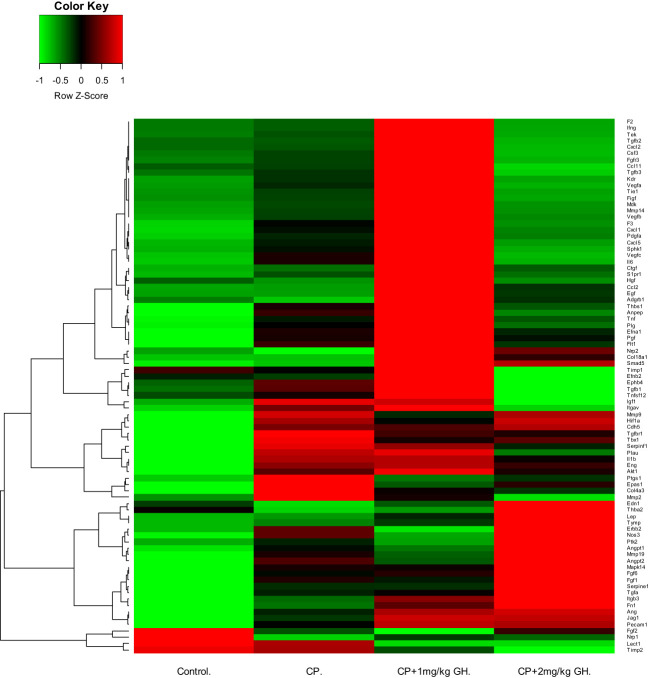
Heatmap image of angiogenesis-related gene expression by quantitative reverse transcription polymerase chain reaction (RT-qPCR), in which up-regulated and down-regulated genes are depicted by green and red, respectively. CP, cyclophosphamide; GH, growth hormone.

**Figure 10:**
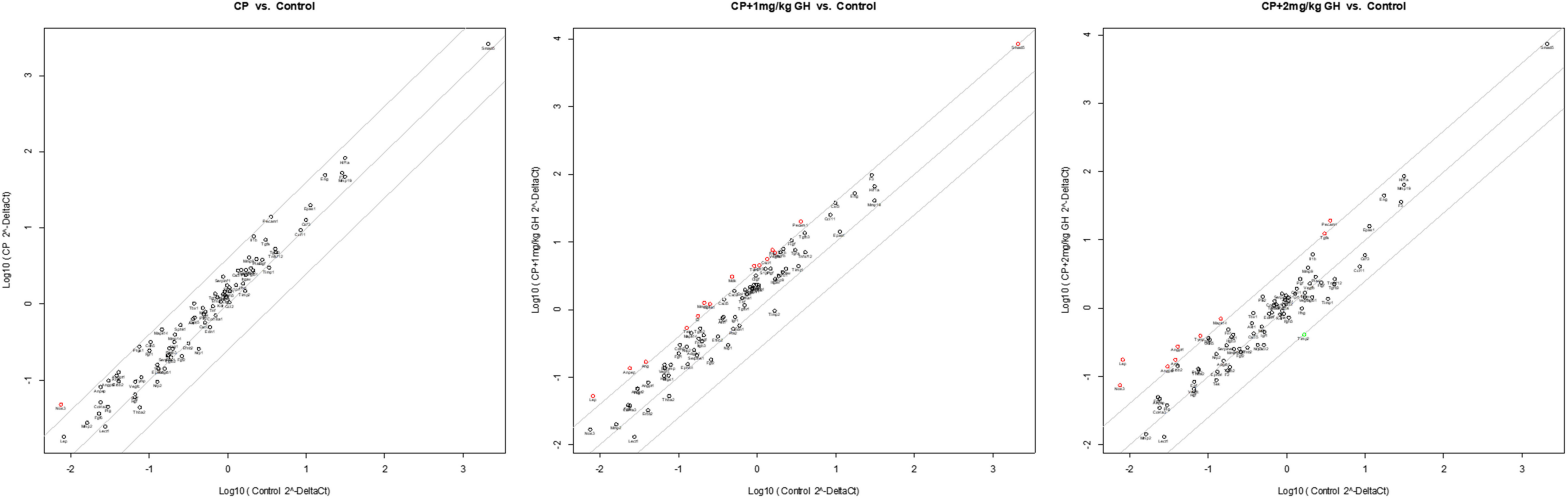
Scatter plot of angiogenesis-related gene expression by quantitative reverse transcription polymerase chain reaction (RT-qPCR). The red dots indicate up-regulation, and the green dots indicate down-regulation. The scatter graph depicts a log transformation plot of the relative expression level of each gene (2^–ΔΔCt^) between control (x-axis) and CP (y-axis) groups. CP, cyclophosphamide; GH, growth hormone.

**Figure 11:**
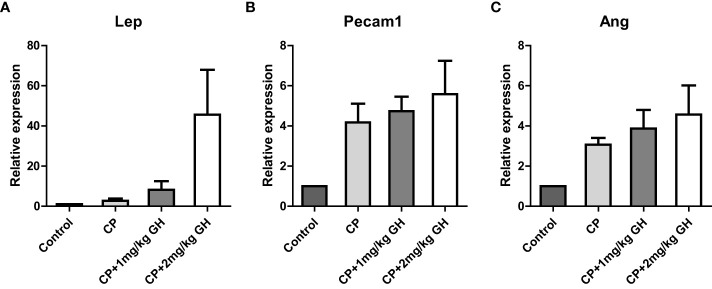
Bar graphs of up-regulated genes showing a >4-fold change in the GH-treated group. The fold changes represent gene expressions compared to those of the control group. **(A)** Leptin (*Lep*), **(B)** platelet endothelial cell adhesion molecule 1 (*Pecam-1*), and **(C)** angiogenin (*Ang*) CP, cyclophosphamide; GH, growth hormone.

A representative image of CD31-immunostained ovaries is shown in **Figure 8A**. CD31, expressed on the surface of endothelial cells, is well established as a maker of vessel density (27). The CD31-positive area was significantly decreased in the CP group compared to that in the control group (**Figure 8B**). The CD31-positive area was significantly increased in the GH-treated group compared with that in the CP group, with no significant differences from that in the control group, indicating restoration of blood vessel density to levels comparable to that in the control group (**Figure 8B**). **Figures 9**, **10** show the expression of 84 angiogenesis-related genes compared to their expression levels in the control group. When generating the heatmap image and scatter plot, the mean CT value obtained from a triplicate experiment was utilized. **Supplementary Table 2** lists the upregulated genes with a > 4-fold change in expression compared with those in the control group. In the GH-treated groups, three genes were upregulated by > 4-fold. These were (leptin [*Lep*] [**Figure 11A**], platelet endothelial cell adhesion molecule 1 [*Pecam-1*] [**Figure 11B**], and angiogenin [*Ang*] [**Figure 11C**]). After selecting these three genes, the mean and standard deviation of the fold change were calculated using CT value obtained from a triplicate experiment, respectively (**Table 2**).

**Table 2:** Up-regulated genes with a fold change value > 4.0 in the GH-treated group.

	Control	CP	CP+1mg/kg GH	CP+2mg/kg GH
Lep	1.00	2.73	8.24	45.62
Pecam1	1.00	4.17	4.73	5.58
Ang	1.00	3.06	3.86	4.57

CP, cyclophosphamide; GH, Growth hormone; Lep, Leptin; Pecam1, Platelet endothelial cell adhesion molecule; Ang, Angiogenin.

**Figure 12:**
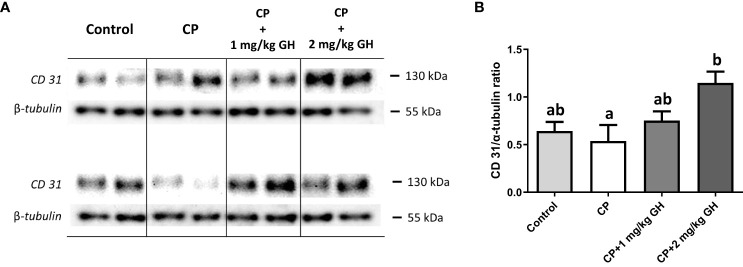
Evaluation of CD31 protein expression by western blot **(A)** Representative western blot image of CD31 **(B)** Relative quantification of CD31. Graphs are presented as mean ± SEM; n=5; different superscript letters indicate statistically significant differences (*p* < 0.05). CP, cyclophosphamide; GH, growth hormone.

CD31 is a protein encoded by the *Pecam-1* gene. Western blot analysis revealed that CD31 expression was significantly increased in the 2 mg/kg GH-treated group compared with that in the CP group (**Figures 12A, B**).

## 4 Discussion

**Figure 13:**
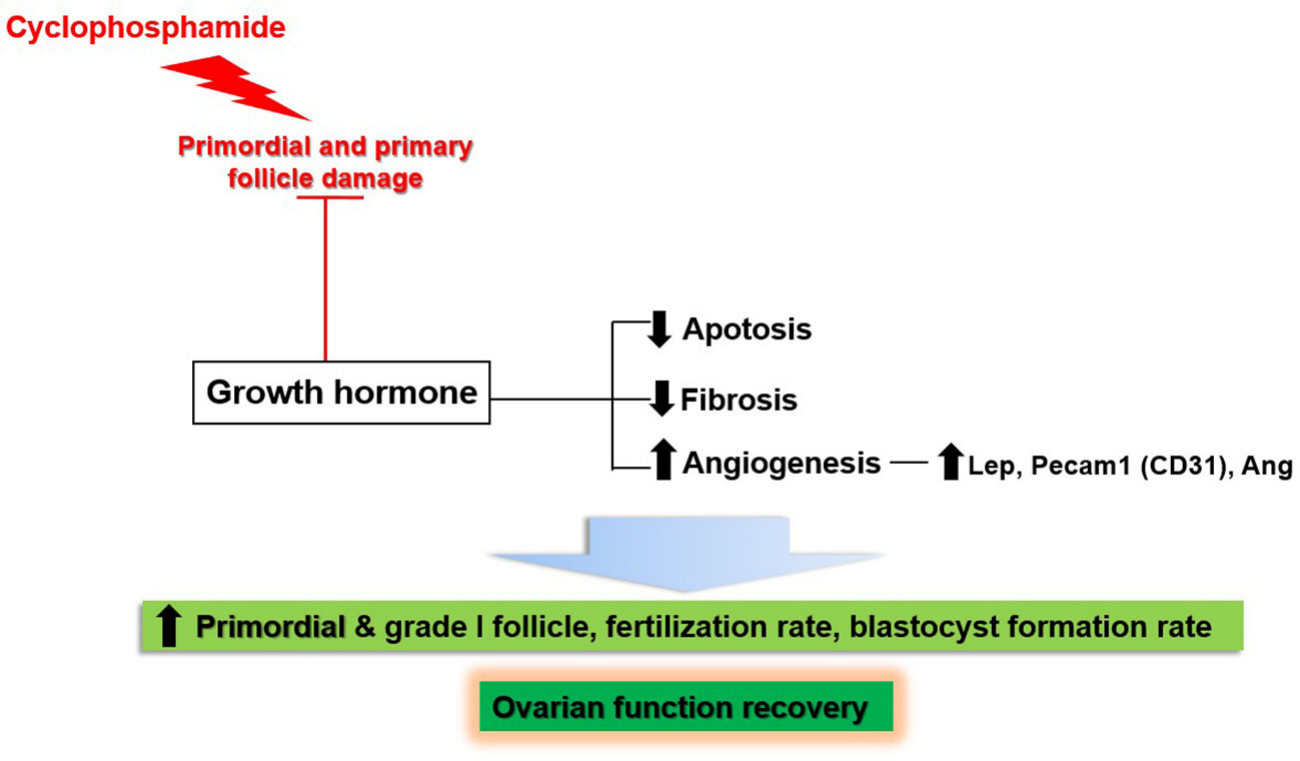
Schematic results of the current study.

This study showed the effects of GH on the recovery of ovarian function in a mouse model of OI induced by the administration of CP. **Figure 13** provides a schematic representation of our results. CP damaged non-growing follicles, including the primordial and primary follicles. Meanwhile, GH administration led to the recovery of ovarian function by decreasing apoptosis and fibrosis; furthermore, it enhanced angiogenesis by upregulation of *Lep*, *Pecam-1*, and *Ang*. As a result, the numbers of primordial and grade I follicles were increased. Furthermore, the fertilization and blastocyst formation rates were increased during IVF.

Anticancer treatment can impact ovarian reserve and potentially induce OI, which increases the risk of infertility, bone loss, and cardiovascular disease. In addition, it impairs self-esteem and causes emotional distress (28). Among patients with breast cancer, those administered CP, particularly at high doses, are considered to have the highest risk of OI (29). CP-based chemotherapy significantly decreases levels of anti-Mullerian hormone and causes treatment-related amenorrhea (1). Various strategies are being developed to enhance ovarian function and overcome these medical hurdles. Among these, GH is gaining increasing interest. Thus, this present study investigated the potential of GH as a therapeutic agent capable of regenerating ovarian function.

GH is a 191 amino acid protein that binds to the growth hormone receptor (GHR). Human oocytes, granulosa cells, and stromal cells express GHR and can be directly influenced by GH (12). GH indirectly affects ovarian function through autocrine and paracrine signaling, mediated by the local production of secondary factors, particularly IGF-I (12). Through these direct and indirect pathways, GH influences folliculogenesis, oocyte maturation, and steroid synthesis (11). GH plays an essential role in the proliferation of ovarian follicles and is necessary for follicular maturation and survival (10). Locally released GH has a major effect on ovarian function, but systemic GH produced by the anterior pituitary gland or from exogenous administration can also have a notable impact on female reproduction (11). GH replacement therapy has been reported to recover ovarian function, allowing successful pregnancies in previously infertile patients with GH deficiency (30). This study demonstrated that GH administration inhibited primordial follicle atresia and increased the number of grade I follicles. The IVF outcomes were also improved with GH administration. Contrary to our expectations, the total number of follicles and the number of grade 1 follicles were not significantly different between the CP and 1 mg/kg GH-treated groups. We speculated that these results could be explained by the small sample size and variation between the mice. Moreover, these results suggest that high-dose GH might be necessary to restore ovarian function.

Our study suggests that the mechanism by which GH restores ovarian function involves the reduction of apoptosis and stromal fibrosis while enhancing angiogenesis levels. Mahran et al. evaluated the radioprotective effects of GH. Immature rats were exposed to single whole-body irradiation (3.2 Gy). They injected 1 mg/kg GH in rats for 7 days, starting from 3 days before irradiation and lasting for 3 days post-irradiation. GH was shown to significantly enhance follicular development and increase serum anti-Mullerian hormone levels. GH also ameliorated oxidative stress-mediated apoptosis by lowering cytochrome c and caspase-3 levels (16). Liu et al. injected 1 mg/kg GH in aged mice (8 months old) every 2 days for 2 months. GH reversed age-associated depletion of ovarian reserve and decline of oocyte quality. Mice in the GH-treated group demonstrated an increase in the average number of estrous cycles, number of follicles, and number of corpus luteum significantly. Further, GH reduced apoptosis by inhibiting Fos and Jun signaling (31). Yigiter et al. evaluated the ovarian tissue-protective effects of GH in a rat model (18). They induced ovarian ischemia and reperfusion injury by clamping or clipping. Thirty minutes before the induction of ischemia or reperfusion injury, 1 mg/kg or 2 mg/kg GH was injected intraperitoneally. GH decreased the number of apoptotic or necrotic cells based on the histopathologic examination and also revealed antioxidant properties. Although those experiments differed in setting from that of the present study, GH may ameliorate apoptosis through various pathways.

To our knowledge, no studies have explored the anti-fibrotic effects of GH against ovarian fibrosis. Fibrosis is characterized by excessive proliferation of fibroblasts and deposition of extracellular matrix. Fibrosis develops in response to repeated tissue injury and inflammation (32); ovarian fibrosis can lead to diminished ovarian function (33). Meirow et al. conducted a case-control study evaluating the ovarian tissue harvested for cryopreservation (34). The case group (n=17) comprised young patients with cancer previously administered chemotherapy, including CP, while the control group (n=18) was not exposed to chemotherapy. Over 75% of case group patients showed ovarian cortical fibrosis with the disappearance of primordial follicles. Meirow et al. also found vascular damage with severe narrowing and obliteration due to intimal fibrosis and hyalinization. These findings were consistent with those of the present study, which identified markedly increased fibrosis and decreased vessel density in the CP group. Meirow et al. suggested the following mechanism for ovarian damage due to chemotherapy: exposure to chemotherapy injures and obstructs blood vessels, causing ischemia of ovarian tissue. Eventually, intact ovarian tissue is replaced by collagenous fibrosis, and primordial follicles are lost. In the present study, fibrosis was similarly significantly reduced in the GH-treated groups; hence, ovarian fibrosis may be reversible with GH treatment, making it a potential therapeutic strategy for enhancing ovarian function.

Angiogenesis refers to the formation of new blood vessels from pre-existing vasculatures by intussusception or sprouting (35). Angiogenesis requires cascade events such as enzymatic degradation of the basal membrane of the pre-existing vessels, migration of endothelial cells, and endothelial cell proliferation (36). As previously mentioned, we found that GH promoted angiogenesis and decreased fibrosis in the damaged ovary. We focused on the angiogenic effects of GH because the supply of GH to the targeted organ requires adequate vascular flow. We did not evaluate the mechanisms by which GH decreases apoptosis and fibrosis. However, we speculated that the observed improvement in angiogenesis contributed to the decrease in apoptosis and fibrosis. This is because angiogenesis is pivotal in maintaining normal ovarian function (37). Adequate blood supply to the ovaries is essential for functions such as supplying nutrients and delivering hormones to the ovary. The female reproductive organs, particularly the ovarian follicles, undergo a regulated angiogenesis process during the emergence of the dominant follicles and early corpus luteum in each menstrual cycle until menopause.

Angiogenesis occurs as an orderly cascade of molecular events starting with angiogenic factor secretion. To understand the mechanism of action of GH on angiogenesis, we analyzed gene expression by RT-qPCR. In this study, GH was found to increase angiogenesis by promoting the secretion of several important angiogenic factors, including Lep, Pecam-1, and Ang.

Lep is an adipocyte-derived peptide hormone that regulates food intake, body mass, and reproductive functions, such as puberty (38). Capillary endothelial cells engage in crosstalk with adipocytes and extracellular components via both paracrine pathways and direct cell-to-cell interactions (39). Lep is also regarded as a potent angiogenic factor. Endothelial cells express the Lep receptor, whereby Lep promotes capillary-like tube formation *in vitro*. Furthermore, Lep increases corneal neovascularization in rats (40). Ovarian follicular cells express Lep receptors, and Lep promotes follicular development and oocyte maturation in synergy with GH and IGF-1 (41).

Pecam-1 (CD31) is a member of the Ig superfamily and is a single-chain transmembrane glycoprotein expressed on endothelial cells, platelets, and leukocytes (42). Pecam-1 has been implicated in the adhesion and signaling cascades required for endothelial cell migration, which plays an essential role in angiogenesis (43). Pecam-1 is also involved in the endothelial cell-to-cell associations required to organize endothelial cells into tubular networks (44).

Ang, a member of the ribonuclease A superfamily, is a potent angiogenic molecule (45). To induce angiogenesis, Ang is first taken up by endothelial cells through receptor-mediated endocytosis, after which it rapidly translocates to the nucleus, where it promotes ribonuclease activity, basement membrane degradation, signaling transduction, and nuclear translocation (46). In addition, the secreted Ang binds to actin on the cell surface to induce basement membrane and extracellular matrix degradation, promoting endothelial cell invasion and migration into the surrounding tissue (47).

The strength of the present study was that our experiments investigated various aspects of the mechanism by which GH restores ovarian function, including apoptosis, fibrosis, and angiogenesis. In addition, RT-qPCR was also conducted to explore the mechanisms by which GH regulates angiogenesis. This study is significant as it is a cornerstone study that used a mouse model to explore the possibility of administering GH to patients with reduced ovarian function before clinical trials can be conducted. To our knowledge, no clinical study has investigated the effects of GH on severely reduced ovarian reserve or premature OI in patients with cancer. However, as demonstrated in this study, GH could effectively restore ovarian function even after cancer treatment. Our findings provide the basis for further studies on the pharmacological recovery of ovarian function. However, the present study has several limitations. First, our mouse model only demonstrated the relatively short-term effects of GH on the recovery of ovarian function. Second, it is unclear whether GH delayed or restored declining ovarian function. Therefore, it will be necessary to compare ovarian function before GH administration with that on the day of ovarian sampling to determine if there is a difference in ovarian function decline. Third, although the GH concentration was determined based on a review of previous studies, we used a very high dose of GH compared to the actual GH concentration typically administered to humans. Fourth, GH could interfere with the activity of chemotherapy. GH may potentially have side effects of metabolic diseases, such as diabetes mellitus (30). Therefore, further studies on the long-term efficacy and safety of GH treatment and the elaboration of the protocol applied in human trials with stringent methodology are recommended.

In conclusion, the present study demonstrated the effects of GH on the recovery of ovarian function in OI induced by the administration of CP. The potential mechanisms by which GH achieved these effects involved decreased apoptosis and stromal fibrosis and increased angiogenesis via upregulation of *Lep*, *Pecam-1*, and *Ang*.

## Data availability statement

The original contributions presented in the study are included in the article/**Supplementary Material**. Further inquiries can be directed to the corresponding authors.

## Ethics statement

The animal study was approved by The experimental protocols were approved by the Institutional Animal Care and Use Committee (IACUC) of Seoul National University Bundang Hospital (BA-2009-304-087-01). The study was conducted in accordance with the local legislation and institutional requirements.

## Author contributions

SK: Conception and design, data interpretation, drafting, and revision of the article. JY: Conception and design, experiment, data analysis, data interpretation, drafting. YH: Conception and design, data interpretation, and revision of the article. JL: Conception and design, data interpretation, revision, and final approval of the article. JK: Conception and design, data interpretation, revision, and final approval of the article. JRL: Conception and design, data analysis, data interpretation, revision, and final approval of the article. All authors contributed to manuscript revision, read, and approved the submitted version.
