# Supplementary material for: The effect of growth hormone on ovarian function recovery in a mouse model of ovarian insufficiency
Source: Front Endocrinol (Lausanne). 2023 Oct 3;14:1184977. doi: 10.3389/fendo.2023.1184977 (PMC10579899; doi:10.3389/fendo.2023.1184977)
Supplement: Supplementary file 1 [file DataSheet_1.docx]

Supplementary Material

Title The effect of growth hormone on ovarian function recovery in a mouse model of ovarian insufficiency

**Su Mi Kim^1,2*^, Jung Young Yoo^3,4*^, Yeon Hee Hong^2,3^, Jaewang Lee^4**^, Ji Hyang Kim^5**^, Jung Ryeol Lee^2,3**^**

*** Correspondence:**

Jung Ryeol Lee: leejrmd@snu.ac.kr

Jaewang Lee: wangjaes@gmail.com

Ji Hyang Kim: bin0902@chamc.co.kr

**
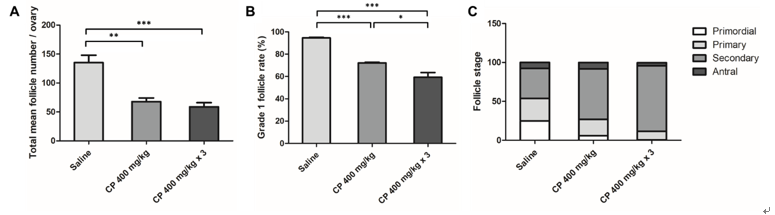
Supplementary Figures**

**Supplementary Figure 1.** Histological analysis of the preliminary study. (A) Total follicle number (B) Grade 1 follicle number, and (C) mean numbers of grade 1 follicles according to the follicular development stages of ovarian tissue. Graphs are presented as mean ± SEM; n=4. ∗*p* < 0.05, ∗∗*p* < 0.01, and ∗∗∗*p* < 0.001. CP: cyclophosphamide

**B**

**A**


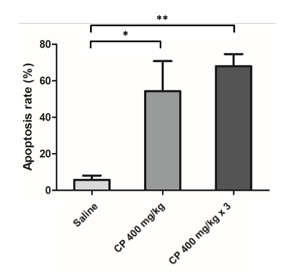
**
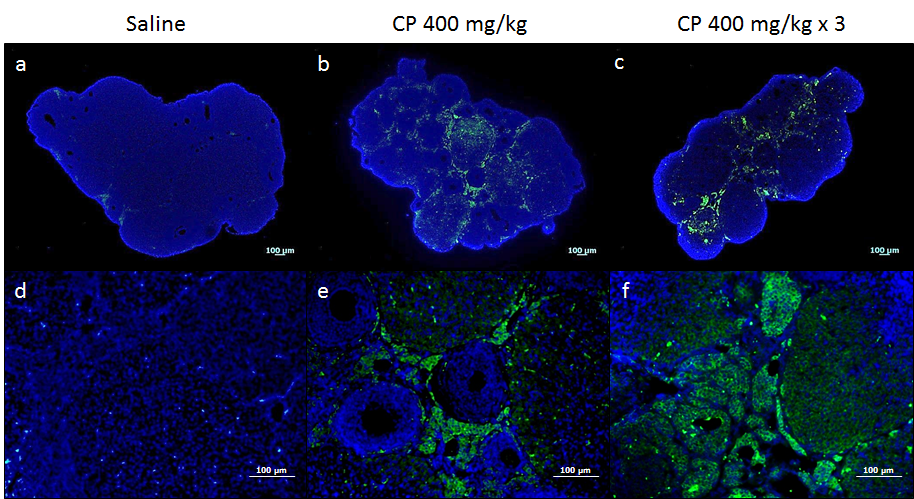
**

**Supplementary Figure 2.** Terminal deoxynucleotidyl transferase dUTP nick-end labeling (TUNEL) assay of the preliminary study. (A) Representative images of TUNEL assay (B) Quantification of the TUNEL positive area. Graphs are presented as mean ± SEM; n=4. ∗p < 0.05, ∗∗p < 0.01, and ∗∗∗p < 0.001. CP: cyclophosphamide

**B**

**A**


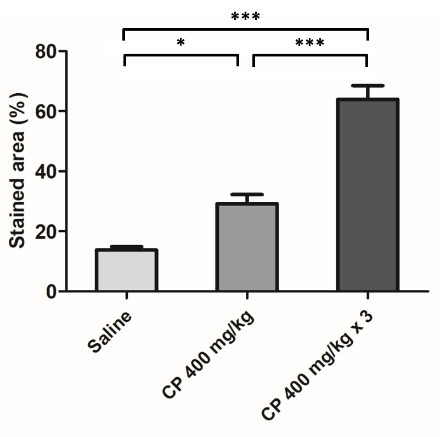

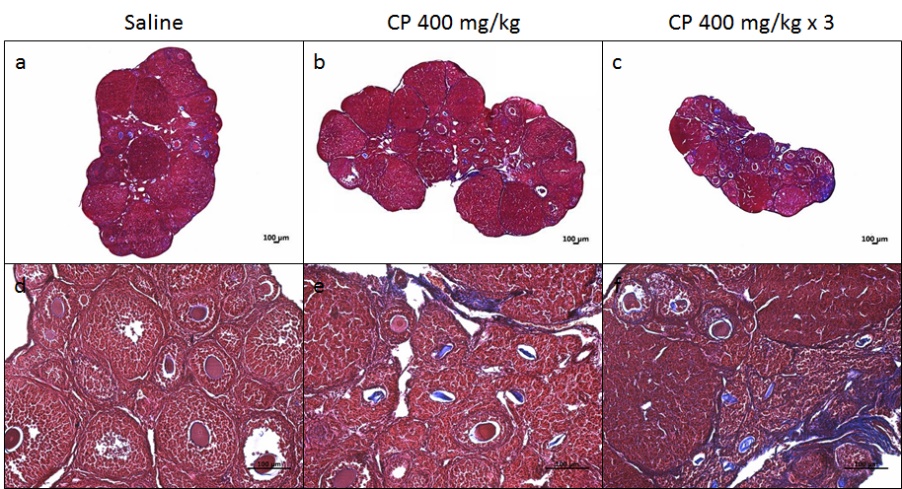


**Supplementary Figure 3.** Masson’s trichrome stain of the preliminary study. (A) The fibrotic surface, nuclei, and cytoplasm were stained blue, black, and red respectively (B) Quantification of the fibrotic area. Graphs are presented as mean ± SEM; n=4. ∗p < 0.05, ∗∗p < 0.01, and ∗∗∗p < 0.001. CP: cyclophosphamide


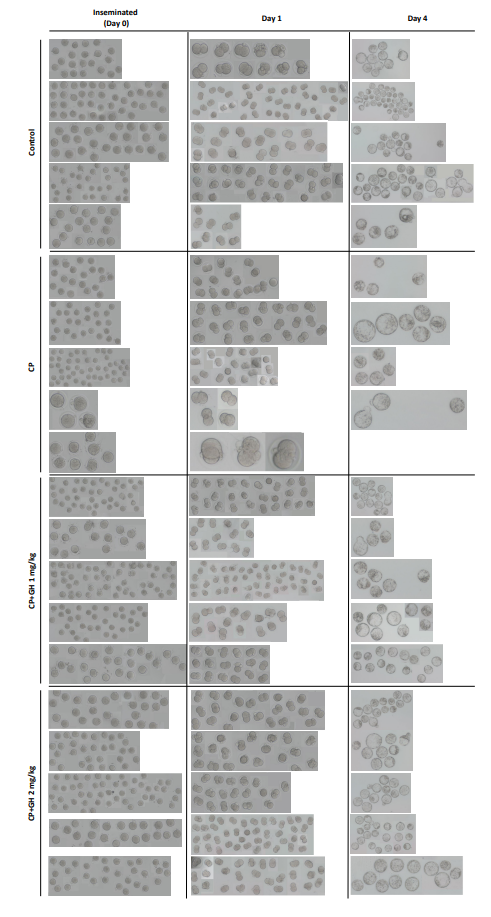


**Supplementary Figure 4.** The retrieved oocytes that developed into blastocysts on day 4. CP: cyclophosphamide; GH: growth hormone.

# Supplementary Tables

**Table 1.** List of genes for Real-time Polymerase Chain Reaction

| **Gene Symbol** | **Gene ID (NCBI)** | **Gene full name** |
| --- | --- | --- |
| Adgrb1 | 107831 | adhesion G protein-coupled receptor B1 |
| Akt1 | 11651 | thymoma viral proto-oncogene 1 |
| Ang | 11727 | angiogenin |
| Angpt1 | 11600 | angiopoietin 1 |
| Angpt2 | 26360 | angiopoietin 2 |
| Anpep | 16790 | Alanyl aminopeptadase, membrane |
| Ccl11 | 20292 | chemokine (C-C motif) ligand 11 |
| Ccl2 | 20296 | chemokine (C-C motif) ligand 2 |
| Cdh5 | 12562 | cadherin 5 |
| Col18a1 | 12822 | collagen, type XVIII, alpha 1 |
| Col4a3 | 12828 | collagen, type IV, alpha 3 |
| Csf3 | 12985 | colony stimulating factor 3 (granulocyte) |
| Ctgf | 14219 | cellular communication network factor 2 |
| Cxcl1 | 14825 | chemokine (C-X-C motif) ligand 1 |
| Cxcl2 | 20310 | chemokine (C-X-C motif) ligand 2 |
| Cxcl5 | 20311 | chemokine (C-X-C motif) ligand 5 |
| Edn1 | 13614 | endothelin 1 |
| Efna1 | 13636 | ephrin A1 |
| Efnb2 | 13642 | ephrin B2 |
| Egf | 13645 | epidermal growth factor |
| Eng | 13805 | endoglin |
| Epas1 | 13819 | endothelial PAS domain protein 1 |
| Ephb4 | 13846 | Eph receptor B4 |
| Erbb2 | 13866 | erb-b2 receptor tyrosine kinase 2 |
| F2 | 14061 | coagulation factor II |
| F3 | 14066 | coagulation factor III |
| Fgf2 | 14173 | fibroblast growth factor 2 |
| Fgf6 | 14177 | fibroblast growth factor 6 |
| Fgf1 | 14164 | Fibroblast growth factor 1 |
| Fgfr3 | 14184 | fibroblast growth factor receptor 3 |
| Figf | 14205 | vascular endothelial growth factor D |
| Flt1 | 14254 | fms related receptor tyrosine kinase 1 |
| Fn1 | 14268 | fibronectin 1 |
| Hgf | 15234 | hepatocyte growth factor |
| Hif1a | 15251 | hypoxia inducible factor 1, alpha subunit |
| Ifng | 15978 | interferon gamma |
| Igf1 | 16000 | insulin-like growth factor 1 |
| Il1b | 16176 | Interleukin 1 beta |
| Il6 | 16193 | Interleukin 6 |
| Itgav | 16410 | integrin alpha V |
| Itgb3 | 16416 | integrin beta 3 |
| Jag1 | 16449 | jagged 1 |
| Kdr | 16542 | kinase insert domain protein receptor |
| Lect1 | 16840 | chondromodulin |
| Lep | 16846 | leptin |
| Mapk14 | 26416 | mitogen-activated protein kinase 14 |
| Mdk | 17242 | midkine |
| Mmp14 | 17387 | matrix metallopeptidase 14 (membrane-inserted) |
| Mmp19 | 58223 | matrix metallopeptidase 19 |
| Mmp2 | 17390 | matrix metallopeptidase 2 |
| Mmp9 | 17395 | matrix metallopeptidase 9 |
| Nos3 | 18127 | nitric oxide synthase 3, endothelial cell |
| Nrp1 | 18186 | neuropilin 1 |
| Nrp2 | 18187 | neuropilin 2 |
| Pdgfa | 18590 | platelet derived growth factor, alpha |
| Pecam1 | 18613 | platelet/endothelial cell adhesion molecule 1 |
| Pgf | 18654 | Placenta growth factor |
| Plau | 18792 | plasminogen activator, urokinase |
| Plg | 18815 | plasminogen |
| Ptgs1 | 19224 | prostaglandin-endoperoxide synthase 1 |
| Ptk2 | 14083 | protein tyrosine kinase 2 |
| S1pr1 | 13609 | sphingosine-1-phosphate receptor 1 |
| Serpine1 | 18787 | serine (or cysteine) peptidase inhibitor, clade E, member 1 |
| Serpinf1 | 20317 | serine (or cysteine) peptidase inhibitor, clade F, member 1 |
| Smad5 | 17129 | SMAD family member 5 |
| Sphk1 | 20698 | Sphingosine kinase 1 |
| Tbx1 | 21380 | T-box 1 |
| Tek | 21687 | TEK receptor tyrosine kinase |
| Tgfa | 21802 | transforming growth factor alpha |
| Tgfb1 | 21803 | transforming growth factor, beta 1 |
| Tgfb2 | 21808 | transforming growth factor, beta 2 |
| Tgfb3 | 21809 | transforming growth factor, beta 3 |
| Tgfbr1 | 21812 | transforming growth factor, beta receptor I |
| Thbs1 | 21825 | thrombospondin 1 |
| Thbs2 | 21826 | thrombospondin 2 |
| Tie1 | 21846 | tyrosine kinase with immunoglobulin-like and EGF-like domains 1 |
| Timp1 | 21857 | tissue inhibitor of metalloproteinase 1 |
| Timp2 | 21858 | tissue inhibitor of metalloproteinase 2 |
| Tnf | 21926 | Tumor necrosis factor |
| Tnfsf12 | 21944 | tumor necrosis factor (ligand) superfamily, member 12 |
| Tymp | 72962 | thymidine phosphorylase |
| Vegfa | 22339 | vascular endothelial growth factor A |
| Vegfb | 22340 | vascular endothelial growth factor B |
| Vegfc | 22341 | vascular endothelial growth factor C |

**Table 2**. Up-regulated genes with a fold change value > 4.0

| **Gene** | **Description** | **Fold Change** |
| --- | --- | --- |
| **CP vs. Control** | | |
| Nos3 | Nitric oxide synthase 3 | 6.42 |
| **CP+1mg/kg GH vs. Control** | | |
| Lep | Leptin | 6.52 |
| Mdk | Midkine | 6.39 |
| Mmp14 | Matrix metalloproteinase 14 | 5.98 |
| Anpep | Alanyl (membrane) aminopeptidase | 5.64 |
| Pecam1 | Platelet endothelial cell adhesion molecule 1 | 5.61 |
| Ifng | Interferon Gamma | 4.91 |
| Sphk1 | Sphingosine kinase 1 | 4.81 |
| Tie1 | Tyrosine kinase with immunoglobulin-like and EGF-like domain 1 | 4.81 |
| Ang | Angiogenin | 4.35 |
| F2 | Coagulation factor ii (thrombin) | 4.56 |
| Tek | Tyrosin-protein kinase | 4.29 |
| Fgfr3 | Fibroblast growth factor receptor 3 | 4.21 |
| Cxcl1 | Chemokine (c-x-c motif) ligand 1 | 4.18 |
| Vegfa | Vascular endothelial growth factor-A | 4.08 |
| **CP+2mg/kg GH vs. Control** | | |
| Lep | Leptin | 21.78 |
| Nos3 | Nitric oxide synthase 3 | 9.93 |
| Angpt1 | Angiopoietin-1 | 6.71 |
| Pecam1 | Platelet endothelial cell adhesion molecule 1 | 5.44 |
| Tymp | Thymidine phosphorylase | 4.94 |
| Mapk14 | Mitogen-activated protein kinase 14 | 4.82 |
| Angpt2 | Angiopoietin-2 | 4.63 |
| Ang | Angiogenin | 4.61 |
| Tgfa | Transforming growth factor-alpha | 4.12 |

CP: cyclophosphamide; GH: Growth hormone.
